# Hypomethylation of GDNF family receptor alpha 1 promotes epithelial-mesenchymal transition and predicts metastasis of colorectal cancer

**DOI:** 10.1371/journal.pgen.1009159

**Published:** 2020-11-11

**Authors:** Zhexu Dong, Lei Dai, Yong Zhang, Chao Fang, Gang Shi, Ye Chen, Junshu Li, Qin Wang, Jiamei Fu, Yan Yu, Wenshuang Wang, Lin Cheng, Yi Liu, Yi Lin, Yuan Wang, Qingnan Wang, Huiling Wang, Hantao Zhang, Yujing Zhang, Xiaolan Su, Shuang Zhang, Feng Wang, Meng Qiu, Zongguang Zhou, Hongxin Deng

**Affiliations:** 1 State Key Laboratory of Biotherapy and Cancer Center, West China Hospital, Sichuan University and Collaborative Innovation Center for Biotherapy, Chengdu, Sichuan, the People’s Republic of China; 2 Department of Gastrointestinal Surgery, West China Hospital and State Key Laboratory of Biotherapy, Sichuan University, Chengdu, Sichuan, the People’s Republic of China; 3 Department of Medical Oncology, Cancer Center, the State Key Laboratory of Biotherapy, West China Hospital, West China Medical School, Sichuan University, Chengdu, Sichuan, the People’s Republic of China; 4 Department of biotherapy, Cancer Center, West China Hospital, Sichuan University and Collaborative Innovation Center for Biotherapy, Chengdu, Sichuan, the People’s Republic of China; 5 Department of Critical Care Medicine, West China Hospital, Sichuan University, Chengdu, Sichuan, the People’s Republic of China; HudsonAlpha Institute for Biotechnology, UNITED STATES

## Abstract

Tumor metastasis is the major cause of poor prognosis and mortality in colorectal cancer (CRC). However, early diagnosis of highly metastatic CRC is currently difficult. In the present study, we screened for a novel biomarker, GDNF family receptor alpha 1 (GFRA1) based on the expression and methylation data in CRC patients from The Cancer Genome Altlas (TCGA), followed by further analysis of the correlation between the GFRA1 expression, methylation, and prognosis of patients. Our results show DNA hypomethylation-mediated upregulation of GFRA1 in invasive CRC, and it was found to be correlated with poor prognosis of CRC patients. Furthermore, *GFRA1* methylation-modified sequences were found to have potential as methylation diagnostic markers of highly metastatic CRC. The targeted demethylation of *GFRA1* by dCas9-TET1CD and gRNA promoted CRC metastasis *in vivo* and *in vitro*. Mechanistically, demethylation of *GFRA1* induces epithelial-mesenchymal transition (EMT) by promoting AKT phosphorylation and increasing c-Jun expression in CRC cells. Collectively, our findings indicate that *GFRA1* hypomethylation can promote CRC invasion via inducing EMT, and thus, *GFRA1* methylation can be used as a biomarker for the early diagnosis of highly metastasis CRC.

## Author summary

Abnormal DNA methylation, one of important characteristics in tumor cells, is exploited as biomarkers for cancer diagnosis and prognosis prediction. Early diagnosis of highly metastatic CRC will be helpful for the clinical management, thus prolongs patient survival. However, it is currently difficult to make early diagnosis of highly metastatic CRC in clinical practice. Currently, we screened a novel biomarker gene, GFRA1, which associated with the invasion and poor prognosis of CRC. The targeted demethylation of GFRA1 exerted a significant promoting effect on CRC metastasis, and GFRA1 methylation-modified sequences are valuable diagnostic biomarker for CRC metastasis risk assessment. Mechanically, demethylation of GFRA1 induced epithelial-mesenchymal transition (EMT) by upregulating AKT phosphorylation and c-Jun expression in CRC cells. Our results demonstrate the promoting effect of *GFRA1* demethylation on CRC invasion and GFRA1 methylation may be a potential prognostic marker for predicting metastasis of CRC.

## Introduction

Colorectal cancer (CRC) ranks third in terms of incidence and second in terms of mortality caused by cancer; more than 1.8 million new CRC cases were diagnosed and 881,000 deaths occurred from CRC in 2018 [1]. Approximately 25% of the CRC patients present metastases at the initial diagnosis stage and almost 50% of them developed metastases, contributing to the high mortality rates associated with CRC [2]. Clinically, colonoscopy is gold standard for the diagnosis of CRC [3, 4]. However, cancer metastases cannot be predicted or identified by the histopathologic or imaging examinations of colonoscopy, there is still lacking effective screening methods for CRC with high metastatic potential [5, 6]. Since invasion of the orthotopic tumors is the first step and the key point in tumor metastasis progression, thus early diagnosis of patients with highly invasive tumor cells can predict the risk of metastasis in CRC [7–9]. Although, the mechanism of invasion of CRC had been investigated previously, a specific and sensitive tumor invasion biomarker capable of predicting CRC metastasis and prognosis in the clinic is lacking [10]. Therefore, developing a novel biomarker for early diagnosis of high metastatic CRC is an effective method to improve the treatment effects and survival of CRC patients.

Substantial evidence has been accumulated suggesting that the epigenome, specifically DNA methylation has a substantial effect on the development of cancer. Therefore, changes in DNA methylation hold great promise as biomarkers for tumor risk prediction [11–13]. In previous study, hypermethylation of DNA measured in blood or stool samples could be used as a biomarker for CRC [14], indicating that DNA methylation biomarkers can be used for CRC clinical diagnosis. As compared to histopathology and imaging examinations, biomarkers can be better predictors of tumor metastasis, including that for CRC [15, 16]. Two methylation-based biomarkers that are located in the GSTpi and Sept9 gene regions are used to predict prostate and colorectal cancers and have been approved by the US FDA, showing that methylated biomarkers have great potential for clinical diagnosis and prediction of cancers [17, 18]. In addition, hypermethylated NEUROG1, RASSF1A, RASSF2A, SDC2, SEPT9, TAC1, and THBD genes were detected in early stage CRC, hypermethylation of ALX4, FBN2, HLTF, P16, TMEFF1, and VIM genes was associated with poor prognosis, and hypermethylated P16 and TFPI2 genes were related to recurrence of CRC [14]. However, few studies on DNA methylation biomarkers associated with CRC invasion or as predictors of metastasis have been reported.

Glial cell derived neurotrophic factor receptor alpha 1 (GFRA1), a member of the GDNF ligand family, plays important roles in nervous system development, spermatogenesis and tumor progression [19]. Previous studies found GFRA1 regulates the proliferation and differentiation of nerve cells [20, 21] and spermatogenic stem cells via binding GDNF ligand [22–24]. Meanwhile, GFRA1 gene can be methylated during spermatogenesis [25]. In addition, GFRA1 can promote the proliferation and migration in pancreatic cancer and breast cancer [26, 27], and induce chemotherapy resistance in osteosarcoma [28]. However, the function and methylation level of GFRA1 in CRC is still not clear.

In order to discover a CRC invasion related DNA methylation biomarker, we used TCGA database data in combination with CRC patient prognostic data to screen for the potential biomarker, which could be associated with CRC tumor invasion and prognosis. GFRA1, a cancer-promoting gene in pancreatic and breast cancers [26, 27], was selected for further analysis. Furthermore, we used dCas9-mediated DNA methylation editing to target demethylation of the GFRA1 gene and investigate the potential function and mechanism of GFRA1 in regulating metastasis in CRC. Our results suggest that *GFRA1* methylation can be used as a biomarker for the early diagnosis of metastasis-risk CRC.

## Results

### Hypomethylation mediated upregulation of GFRA1 correlated with invasion and poor prognosis of CRC

**Fig 1:**
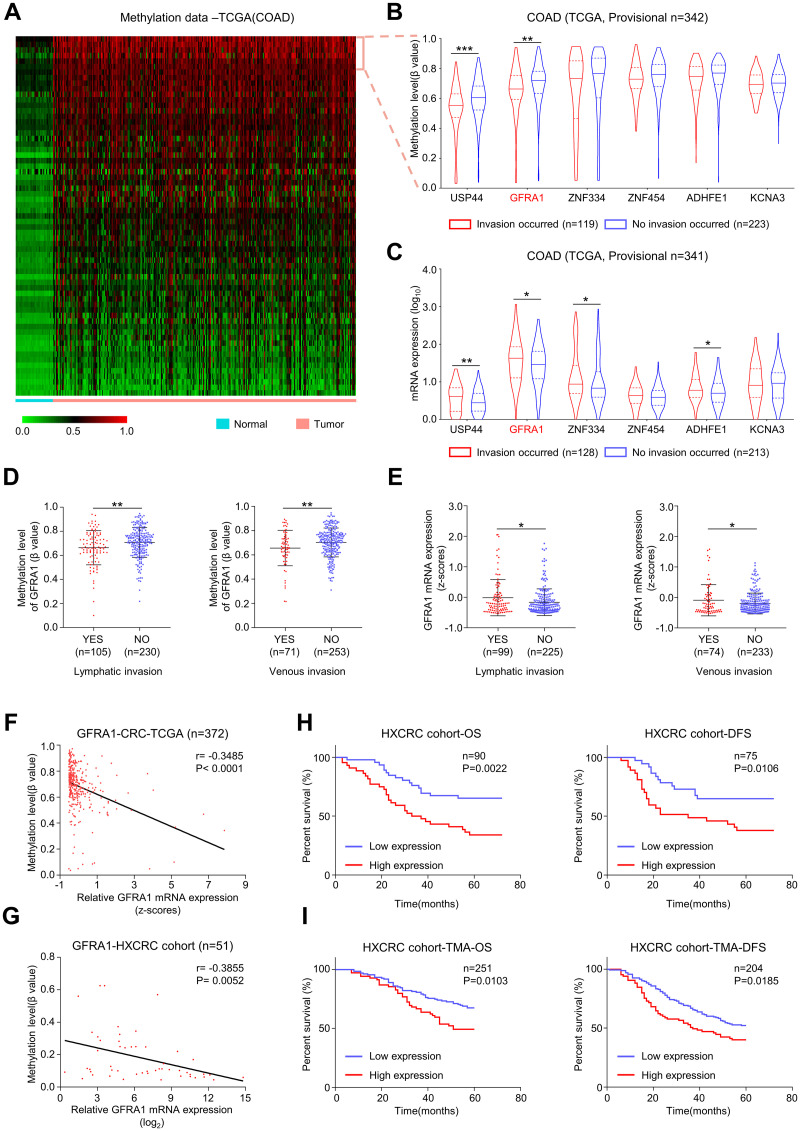
Hypomethylation mediated upregulation of GFRA1 in colorectal cancer is associated with tumor invasion and poor prognosis. **A** Heat map showing associated with the methylation profile of 65 genes in normal and tumor tissues from S1A Fig, β value indicating the DNA methylation levels are represented by the color of the heat map(Red means high methylation, green means low methylation). **B** Violin plot of differentially methylated genes in CRC tumors with or without invasion based on TCGA (**p < 0.01, ***p < 0.001; student t-test). **C** Violin plot of differentially expression level of methylated genes in CRC tumors with or without invasion based on TCGA (*p < 0.05, **p < 0.01; student t-test). **D** Comparison the GFRA1 gene methylation in tumors with or without lymphatic invasion and venous invasion from TCGA (**p < 0.01; student t-test). **E** Comparison the GFRA1 mRNA expression (z-scores relative to diploid samples) in tumors with or without lymphatic invasion and venous invasion from TCGA (*p < 0.05; student t-test). **F** Correlation analysis between GFRA1 gene expression (z-scores relative to diploid samples) and methylation levels from TCGA (Pearson’s correlation test). **G** Correlation analysis between GFRA1 gene expression and methylation levels from HXCRC cohort patient samples (Pearson’s correlation test). **H** Overall and disease-free survival of patients from HXCRC cohort GFRA1 RNA expression data (log-rank (Mantel-Cox) test). Data are mean ± SD. **I** Overall and disease-free survival of patients from HXCRC cohort GFRA1 protein expression data (IHC staining in TMA, log-rank (Mantel-Cox) test). Data are mean ± SD.

To screen for potential genes that were regulated by methylation in CRC, we analyzed the RNA-seq data and methylation data in 38 normal tissues and 309 Colon adenocarcinoma (COAD) tumor tissues from The Cancer Genome Atlas (TCGA), in which 65 hypermethylated genes with significant down-regulation of RNA levels were found (S1A and S1B Fig, Fig 1A). After that, we compared the methylation and mRNA expression level of candidate genes between invasive and non-invasive CRC tumors. Among these genes, GFRA1 was screened for its significant hypomethylation and high expression in invasive CRC compared to non-invasive colon cancer (Fig 1B and 1C). Furthermore, the clinical relevance of GFRA1 in CRC was determined based on the TCGA database. The results suggested that GFRA1 methylation levels were significantly reduced and the mRNA levels were significantly elevated in patients with lymphatic invasion and vascular invasion (Fig 1D and 1E) Next, correlation analysis from TCGA and HXCRC cohort showed that the methylation levels of GFRA1 were slight negatively correlated with the gene expression (Fig 1F and 1G). The treatment of CRC cell lines with anti-methylation drug DAC could significantly increase the expression of GFRA1 (S1C–S1E Fig), this indicates that GFRA1 expression could be upregulated by GFRA1 demethylation. Then, the GFRA1 mRNA expression was determined in a HXCRC cohort containing 90 malignant tissues of CRC patients. The analysis indicated that the high expression of GFRA1 is consistently correlated with shorter overall survival and disease-free survival (Fig 1H). Furthermore, we also got similar results on the HXCRC cohort tissue microarrays (TMA) containing 251 malignant tissues of CRC patients based on the GFRA1 protein expression (Fig 1I, S1F Fig). Taken together, these results suggested that hypomethylation -mediated GFRA1 upregulation associated with metastasis and poor prognosis of CRC patients.

### *GFRA1* hypomethylation associates with tumor invasion and poor prognosis in CRC

**Fig 2:**
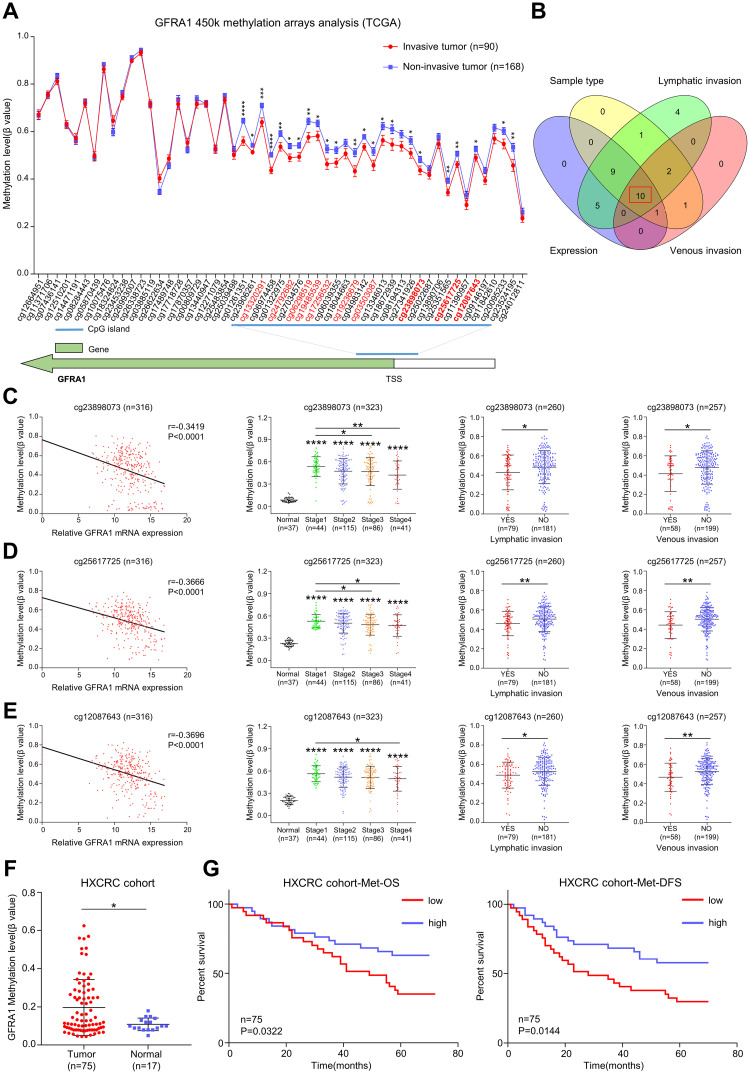
Analysis of full-length methylation modification of GFRA1 gene in CRC based on 450K methylation microarray. **A** Full-length 450K methylation array data analysis of GFRA1 gene in invasive CRC tumors compared to non-invasive CRC tumors (*p < 0.05, **p < 0.01, ***p < 0.001, ****p < 0.0001; student t-test, Data are mean ± SEM). **B** Venn diagram used to demonstrate a common list of 10 methylated sequences. Including the correlation between the probe sequences DNA methylation level and gene expression (r < -0.3; Pearson’s correlation test, Expression), the methylation modification of probe sequences in CRC (p < 0.05; student t-test, Sample type), significant reduction of DNA methylation level in lymphatic invasive CRC (p < 0.05; student t-test, Lymphatic invasion)and vascular invasive CRC (p < 0.05; student t-test, Venous invasion). marked in red (A). **C-E** Detailed description of three methylation sequences upstream of GFRA1 gene TSS from (B), including correlation between gene expression and DNA methylation (Pearson’s correlation test), variety in DNA methylation levels at different tumor stages (*p < 0.05, **p < 0.01, ****p < 0.0001; student t-test) and differences in methylation levels between invasive and non-invasive tumors (*p < 0.05, **p < 0.01; student t-test). **F** Comparison of the methylation levels of *GFRA1* TSS upstream in CRC tumor tissues and normal tissues from HXCRC cohort (*p < 0.05; student t-test). **G** Overall and disease-free survival of patients from HXCRC cohort methylation data in (F) (log-rank (Mantel-Cox) test). Data are mean ± SD.

To further explore the relationship between *GFRA1* methylation and tumor invasion, we performed a full-length methylation modification analysis of *GFRA1* using 450K methylation chip data derived from TCGA. As compared to the non-invasive tumor tissues, the significantly hypomethylated region in the invasive tumor was mainly located in the CpG island of the transcription start site (TSS) (Fig 2A). Combining clinical invasion phenotype and *GFRA1* expression correlation analysis, we found 10 eligible probe sequences (Fig 2B and S1 Table). Among them, three sequences are located upstream of the TSS, and seven sequences are located downstream of the TSS (Fig 2A). In further analysis, the three probe sequences upstream of TSS were found to be negatively correlated with the gene expression (Fig 2C–2E). The methylation level of this sequence was also significantly reduced in stage 3–4 colon cancer tissues, and in tumor tissues with lymphatic invasion and vascular invasion (Fig 2C–2E). The other seven probe sequences downstream of TSS were found to have similar results (S2 Fig). To further evaluate the performance of *GFRA1* methylation in predicting CRC invasion, ROC curves were initially calculated using probe sequence data with significantly reduced methylation levels (P<0.001) in lymphatic invasive tumors. As shown in S3 Fig, the AUC values of the eight probe sequences ranged from 0.6 to 0.7, indicating that *GFRA1*-low methylation can be used as a methylation biomarker for tumor invasion. After that, we used 92 clinical patient samples (75 tumor tissues and 17 paracancerous tissues) to determine the degree of methylation upstream of *GFRA1* TSS by time-of-flight mass spectrometry. The methylation sites around the cg25617725 probe sequence have higher methylation modification in colon cancer patients (Fig 2F), but hypomethylated patients with shorter overall survival and disease-free survival (Fig 2G). In summary, *GFRA1* epigenetic modification sequences in TSS associated with tumor invasion and poor prognosis.

### dCas9-TET1CD mediated *GFRA1* specific demethylation

**Fig 3:**
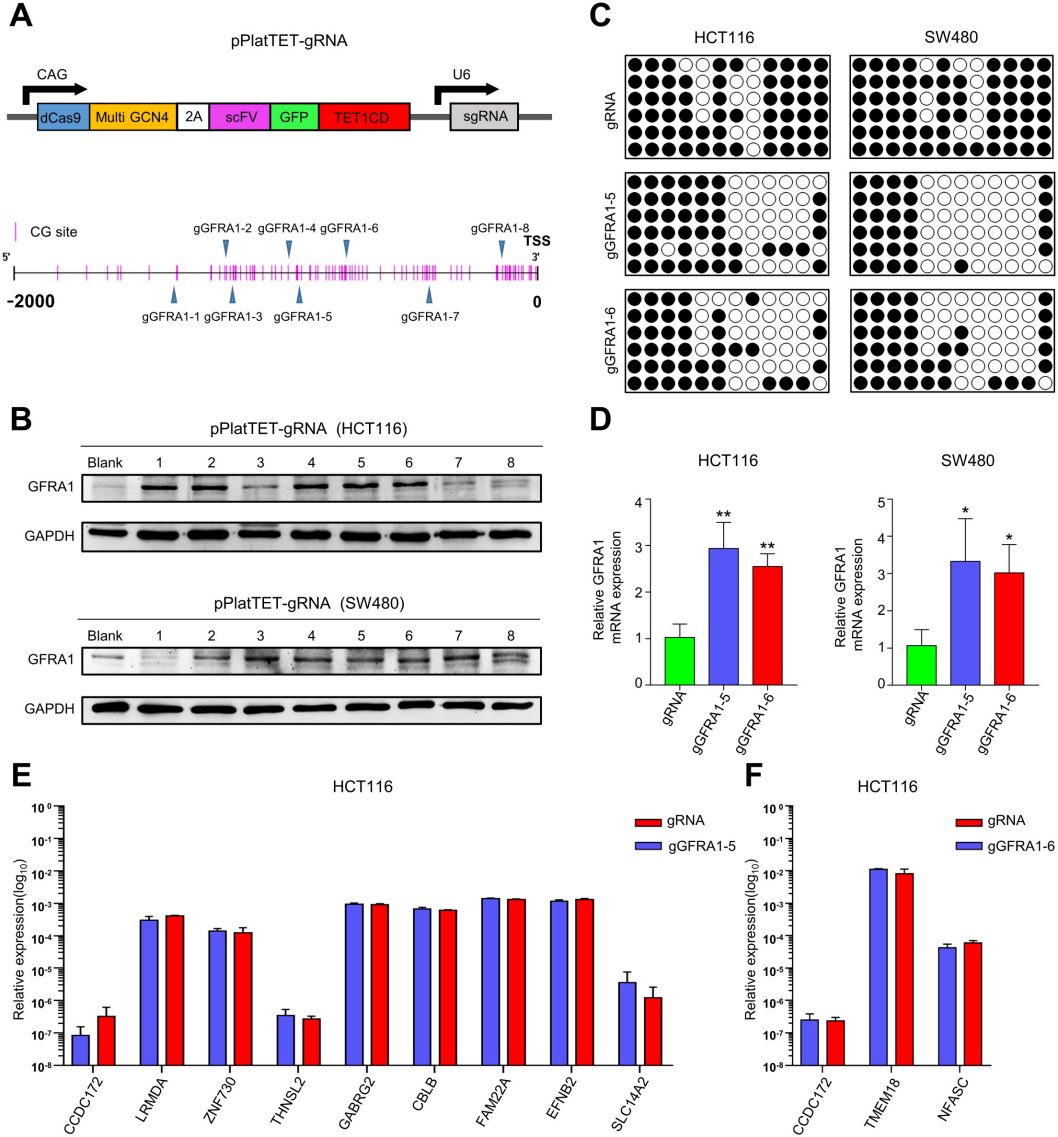
Targeted demethylation of *GFRA1* by dCas9-Tet1CD and verification of off-target effect in CRC cells. **A** Schematic representation of dCAS9-mediated targeted demethylation plasmid and distribution of gRNA the upstream of the *GFRA1* TSS. **B** Expression of GFRA1 after transfection of pPlatTET-gRNA plasmids constructed from different gRNAs in HCT116 and SW480 cells was detected using western blotting. **C** BSP-seq determination of TSS region demethylation effect of pPlatTET-gGFRA1-5 and pPlatTET-gGFRA1-6 in HCT116 and SW480 cells (methylation CpG sites are shown as black dots, unmethylation CpG sites are shown as white dots). **D** The mRNA levels of GFRA1 in *GFRA1*-targeted demethylation CRC cells detected by Real-time PCR analysis (*p < 0.05, **p < 0.01; student t-test). **E-F** The relative expression of genes at gGFRA1-5 and gGFRA1-6 off-target sites in HCT116 cells (Student’s t-test). Data are mean ± SD.

To test whether demethylation of the hypermethylated TSS can reactivate *GFRA1*, we constructed gRNA-mediated *GFRA1*-targeted methylation editing plasmid (Fig 3A). This plasmid recruits dCas9-TET1CD to the TSS of *GFRA1 via* gRNA [29], thus specifically demethylating the TSS region of *GFRA1*. We found that most of the *GFRA1* gene in CRC cell lines has high methylation modifications in the CCLE database (S4A Fig). Therefore, HCT116 and SW480 cells, which show hypermethylation of *GFRA1* by BSP sequencing (S4B Fig), were used for targeted *GFRA1* demethylation. We designed eight gRNAs and selected the two best gRNAs (gGFRA1-5 and gGFRA1-6) based on the GFRA1 protein expression in western blotting (Fig 3B, S2 Table), Furthermore, we subsequently proved that dcas9-TET1CD-gGFRA1-5 and dcas9-TET1CD-gGFRA1-6 significantly removed the *GFRA1* gene methylation and upregulated the GFRA1 mRNA expression in HCT116 and SW480 cells (Fig 3C and 3D). Since our DNA targeting methylation editing system is mediated through the use of gRNA, it was required to determine whether gene-targeted demethylation was off target. By using the gRNA off-target sequence prediction website (https://crispr.bme.gatech.edu/), we identified the 10 most likely off-target sites for gGFRA1-5 and gGFRA1-6 (S3 and S4 Tables). Among them, the gGFRA1-5 off-target site corresponds to 9 genes (CCDC172, LRMDA, ZNF730, THNSL2, GABRG2, CBLB, FAM22A, EFNB2, and SLC14A2), and the gGFRA1-6 off-target site corresponds to 3 genes (CCDC172, TMEM18, and NFASC). We further used qRT-PCR to detect gene expression of the gRNA off-target sites after transfection of dcas9-TET1CD-gGFRA1-5 and dcas9-TET1CD-gGFRA1-6 in HCT116 and SW480 cells. It was found that *GFRA1*-targeted methylation editing plasmid did not cause significant changes in the off-target gene expression (Fig 3E and 3F and S4C and S4D Fig). Collectively, dcas9-TET1CD-gGFRA1-5 and dcas9-TET1CD-gGFRA1-6 could induce targeted and specific demethylation of *GFRA1* in CRC.

### Targeted demethylation of *GFRA1* promotes CRC metastasis

**Fig 4:**
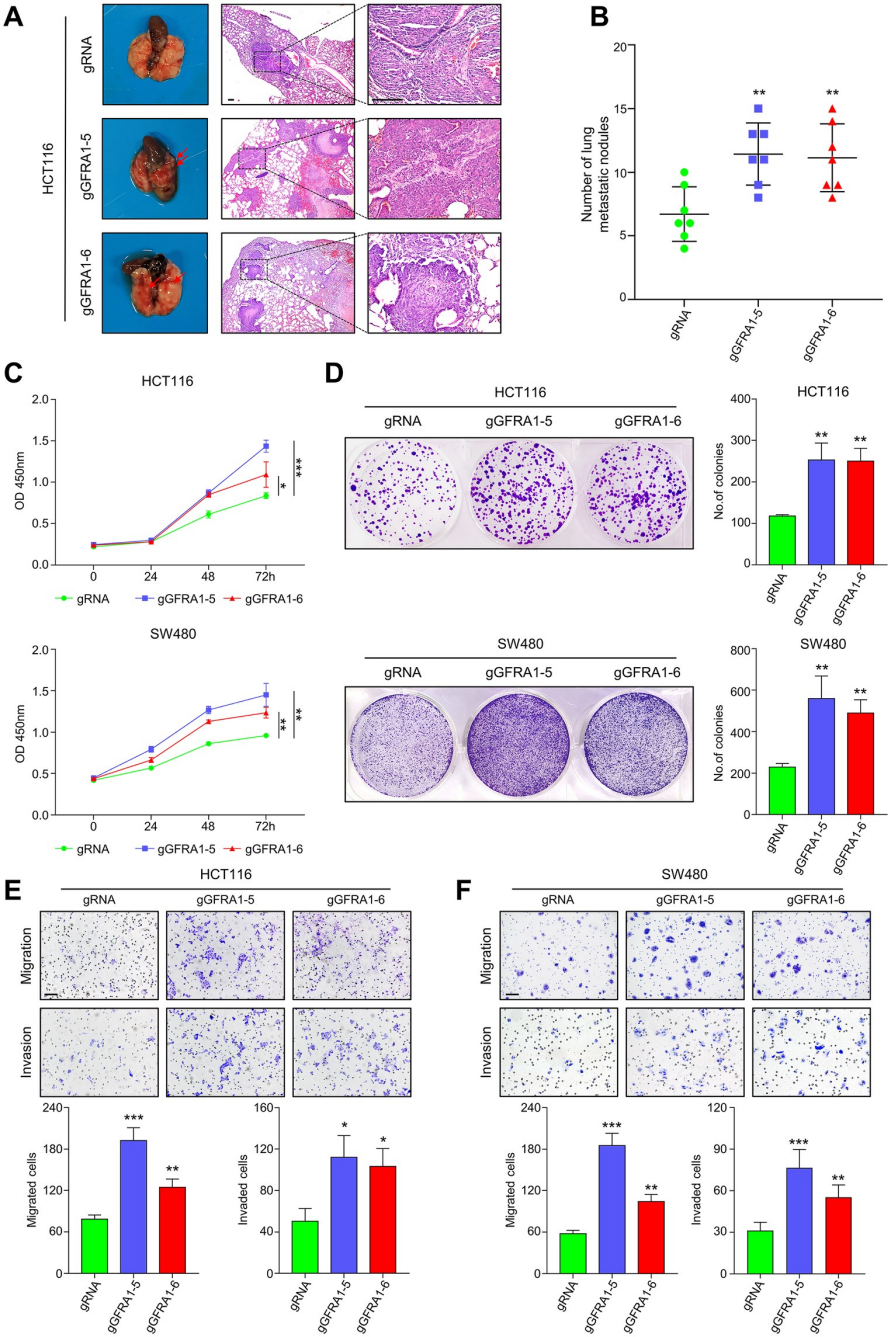
Hypomethylation of *GFRA1* promotes metastasis of CRC. **A-B** Female BALB/c nude mice were injected in tail vein with either HCT116 cells with targeted demethylation of *GFRA1* (pPlatTET-gGFRA1-5 and pPlatTET-gGFRA1-6) or respective control cells (pPlatTET-gRNA), n = 7 mice per group. Representative hematoxylin and eosin-stained images presented to show tumor lesions in the lungs (scale bar, 100 μm). The scatter plots show the number of lesions in the lungs as mean± SD (**p < 0.01; Student’s t-test). **C** CCR8 experiment demonstrates the effect of *GFRA1* demethylation on cell proliferation in HCT116 and SW480 cells (*p < 0.05, **p < 0.01, ***p < 0.001; Student’s t-test). **D** Effects of the loss of *GFRA1* methylation on clonogenic spheroid formation in CRC cells was detected by clonogenic spheroid formation assay (**p < 0.01; student t-test). **E-F** Effects of *GFRA1* hypomethylation on cell migration and invasion, as evaluated by Transwell assays in CRC cells (*p < 0.05, **p < 0.01, ***p < 0.001; Student’s t-test), and data are mean ± SD from at least three independent experiments. Scale bars represent 100 μm.

To test whether *GFRA1* hypomethylation will promotes CRC metastasis, HCT116 cells after transfection with dcas9-TET1CD-gGFRA1-5 or dcas9-TET1CD-gGFRA1-6 were injected into the tail vein of BALB/c nude mice. One month after HCT116 cell injection, the organ metastases in nude mice were determined. As shown in Fig 4A and 4B, more lung metastasis nodules were observed in the groups with *GFRA1* hypomethylation, as compared to the control group. Next, we evaluated the ability of *GFRA1* targeted demethylation on the proliferative capacity of HCT116 and SW480 cells using CCK8 proliferation assay and colony formation assay *in vitro*. The results indicated that the proliferative capacity of HCT116 cells and SW480 cells was significantly enhanced after removal of *GFRA1* methylation (Fig 4C). Similarly, the colony formation assay suggested that the targeted demethylation of GFRA1 also significantly increased the number of clones produced by CRC cells after two weeks (Fig 4D). Moreover, the effect of *GFRA1* hypomethylation on the motility and invasion capability of CRC cells *in vitro* was detected in Transwell assay. After incubating for 48 h, the *GFRA1*-targeted demethylation significantly increased the migration and invasion of HCT116 and SW480 cells (Fig 4E and 4F). These results suggest that *GFRA1* hypomethylation may serve as a tumor promoting factor to induce the metastasis of CRC, as shown in previous clinical data analysis results.

### Targeted demethylation of GFRA1 gene induces epithelial to mesenchymal transition in CRC cells

**Fig 5:**
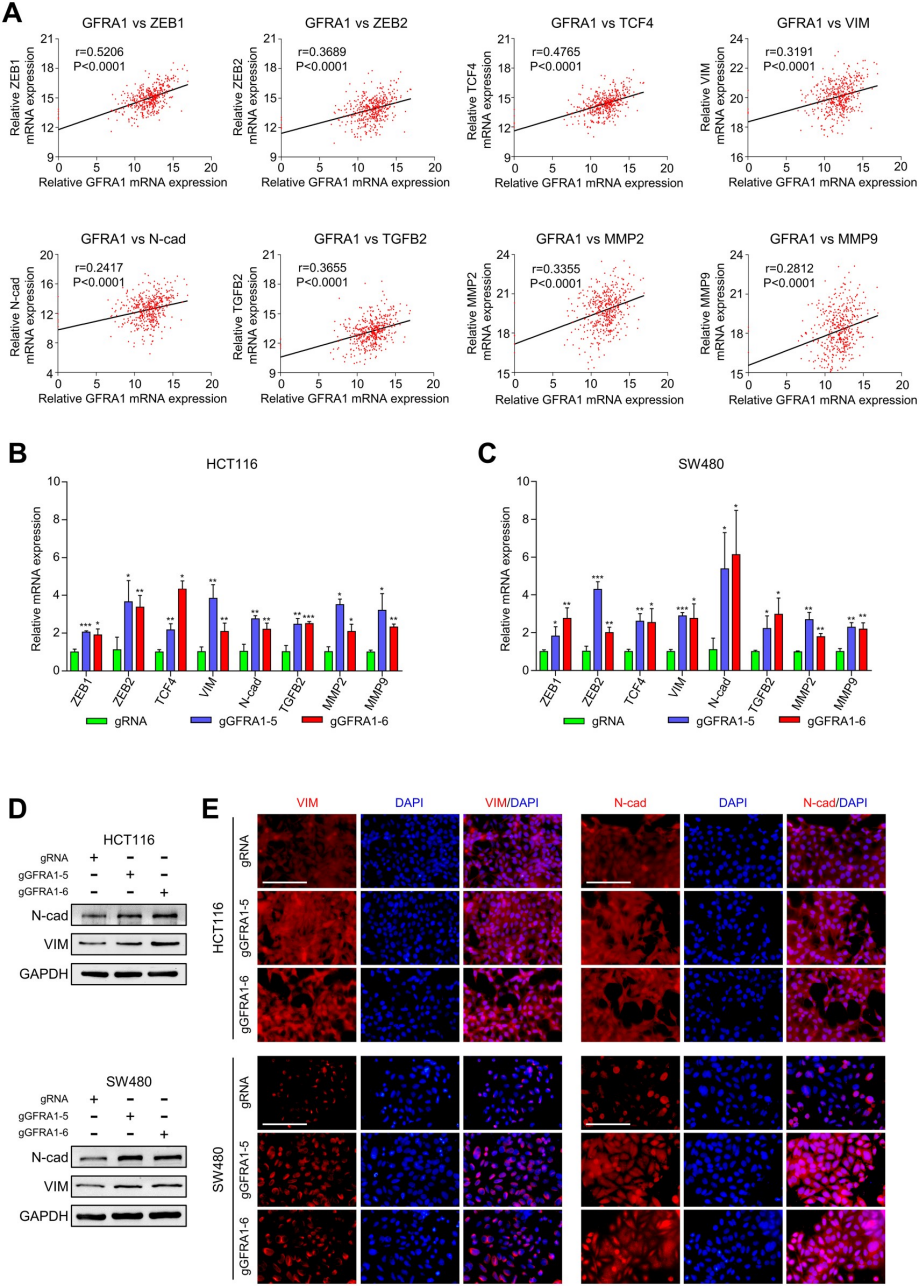
*GFRA1* hypomethylation activates EMT in CRC cells. **A** Gene co-expression analysis showing the correlation of GFRA1 to ZEB1, ZEB2, TCF4, VIM, N-cad, TGFB2, MMP2, and MMP9 (Pearson correlation test). **B-C** q-PCR assays display the effects on gene expression (A) in GFRA1 hypomethylation CRC cells (*p < 0.05, **p < 0.01, ***p < 0.001; Student’s t-test). **D** Western blot analyses showing the effects of *GFRA1*-targeted demethylation on the expression of EMT marker (VIM and N-cad) proteins in CRC cells. **E** Immunofluorescence assays depicting localization and protein expression of EMT markers after *GFRA1* demethylation. Data are mean ± SD from at least three independent experiments. Scale bars represent 100 μm.

To further explore the mechanism of *GFRA1* targeted demethylation in promoting CRC metastasis, we performed gene co-expression analysis by using TCGA database data. The results demonstrated a slight positive correlation between GFRA1 mRNA expression and EMT-activating transcription factors (ZEB1, ZEB2, SNAI2, and TCF4), EMT-markers (VIM, N-cad), invasion-related factors (MMP2 and MMP9), and growth factors (TGFB2, IGF1, FGF2, and VEGFC) (Fig 5A, S5A Fig). However, there was no significant correlation between SNAI1, TWIST, CDH1 (E-cad), VEGFA, MMP7, and GFRA1 mRNA levels in patient samples from TCGA (S5A Fig). Next, we examined whether the expression of these positively related genes were regulated by *GFRA1* methylation in CRC cells. As shown in Fig 5B and 5C, the mRNA expression of ZEB1, ZEB2, TCF4, VIM, N-cad, TGFB2, MMP2, and MMP9 was significantly increased with the target-demethylation of *GFRA1* in HCT116 and SW480 cells. In contrast, the IGF1, FGF2, VEGFC, and SNAI2 mRNA levels did not change evidently (S5B and S5C Fig), indicating that the expression of these genes is not regulated by GFRA1 expression. Moreover, we examined the protein expression levels of EMT markers in *GFRA1* targeted demethylated CRC cells. As shown in Fig 5D, the mesenchymal markers, VIM and N-cad were upregulated in CRC cells with *GFRA1* hypomethylation. Furthermore, immunofluorescence analysis also showed an increase in VIM and N-cad expression in *GFRA1* targeted demethylation HCT116 and SW480 cells, as compared to that in the control cells (Fig 5E). Taken together, our data indicated that *GFRA1* hypomethylation promoted metastasis through the induction of the EMT program in CRC cells.

### Hypomethylation of *GFRA1* induces epithelial to mesenchymal transition by promoting AKT phosphorylation and upregulating c-Jun expression in CRC

**Fig 6:**
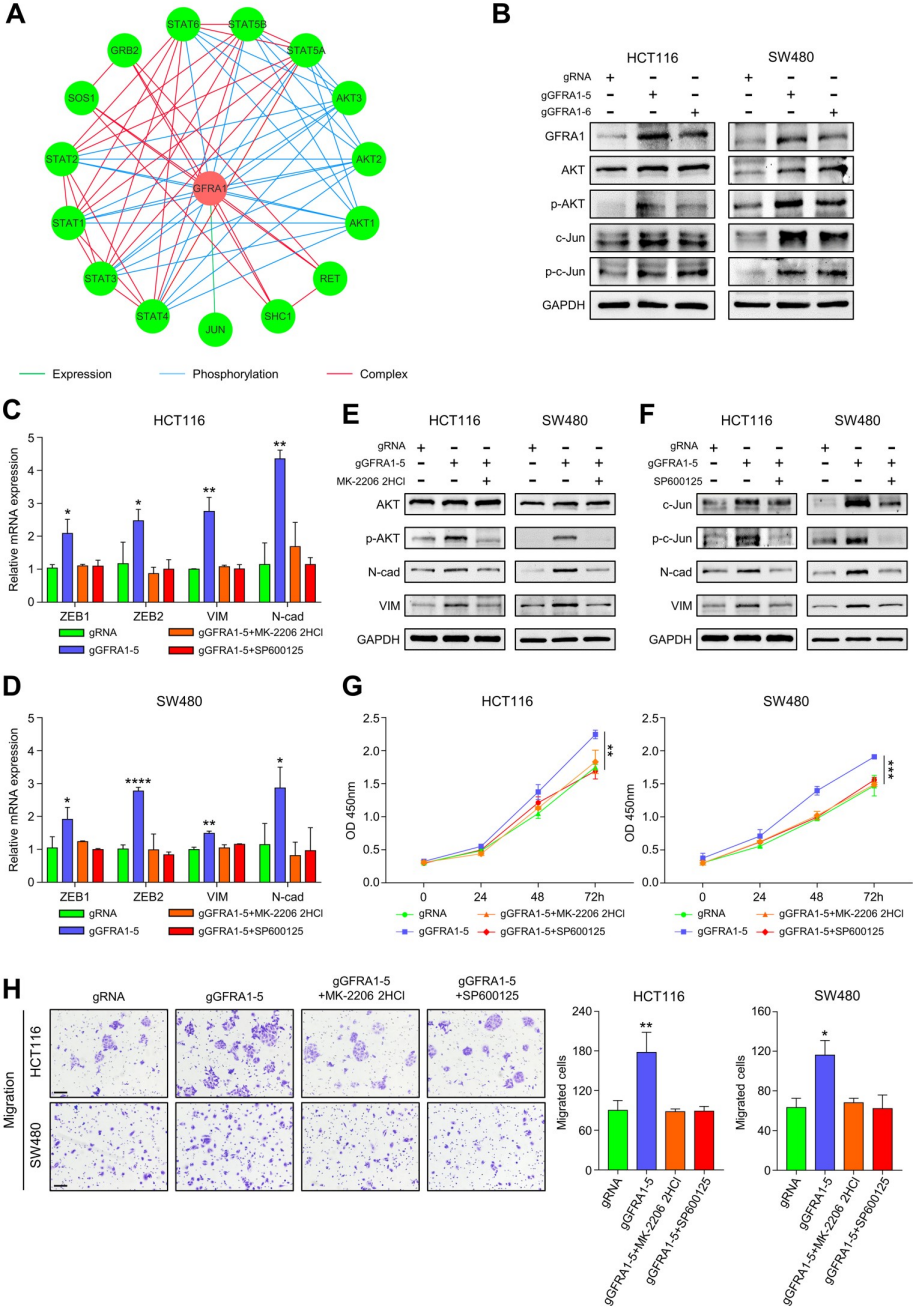
Hypomethylation of *GFRA1* leads to EMT being blocked by p-AKT and p-C-Jun inhibitors. **A** Pathway Common network conducted by GFRA1 related genes. The nodes represent genes, edges display an interaction between query genes (Green line: control expression; Blue line: control Phosphorylation; Red line: in complex). **B** Western blot analyses display the effects of *GFRA1* hypomethylation on the protein expression of AKT, p-AKT, c-Jun, and p-c-Jun in HCT116 and SW480 cells. **C-D** The mRNA expression levels of ZEB1, ZEB2, VIM, and N-cad in *GFRA1* hypomethylation CRC cells treated with MK-2206 2HCl and SP600125 or DMSO, were detected by q-PCR (*p < 0.05, **p < 0.01, ****p < 0.0001; Student’s t-test). **E-F** Western blot analyses showing the protein expression of AKT, p-AKT, c-Jun, and p-c-Jun in HCT116-gGFRA1-5 and SW480-gGFRA1-5 cells treated with MK-2206 2HCl (E) and SP600125(F) or DMSO. **G** CCK8 experiment demonstrates the effect of treatment with MK-2206 2HCl and SP600125 on cell proliferation in *GFRA1* targeted demethylation cells (**p < 0.01, ***p < 0.001; Student’s t-test). **H** Effects of MK-2206 2HCl and SP600125 treatment on cell migration in *GFRA1* hypomethylation CRC cells, as evaluated by Transwell assays. (*p < 0.05, **p < 0.01; Student’s t-test), and the data are mean ± SD from at least three independent experiments. Scale bars represent 100 μm.

To elucidate the mechanism of EMT induced by *GFRA1* hypomethylation in CRC cells, we performed GFRA1 related pathway commons network analysis by using data from the public pathway and interactions databases (http://www.pathwaycommons.org/). The *GFRA1* gene was found to be associated with AKT phosphorylation and expression of Jun in CRC (Fig 6A). Then, western blotting analysis showed that the protein expression levels of p-AKT, c-Jun, and p-c-Jun were much higher in the *GFRA1*-hypomethylatedd cells than in their respective control cells (Fig 6B). To determine whether *GFRA1* demethylation induces EMT by promoting AKT and c-Jun phosphorylation in CRC, the AKT inhibitor MK-2206 2HCl and JNK inhibitor SP600125 were added at a concentration of 2 μM for 48 h to the *GFRA1* targeted demethylation CRC cells. The mRNA expression levels of ZEB1, ZEB2, VIM, and N-cad were decreased after treatment of *GFRA1*-hypomethylation cells with MK-2206 2HCl and SP600125 at 2 μM for 24 h (Fig 6C and 6D). Moreover, the protein expression levels of VIM and N-cad were lower with the reduction of p-AKT and p-c-Jun proteins in *GFRA1*-target demethylation HCT116 and SW480 cells, which was consistent with the mRNA expression data (Fig 6E and 6F). In addition, the proliferation and migration of SW480-*GFRA1* and HCT116-*GFRA1* hypomethylated cells were reduced by treatment with the inhibitors, MK-2206 2HCl and SP600125 (Fig 6G and 6H), suggesting that the promotion of *GFRA1* hypomethylation in the CRC cell proliferation and invasion can be blocked by p-AKT and p-c-Jun inhibitors. All of these results demonstrate that hypomethylation of *GFRA1* promotes AKT phosphorylation and upregulates c-Jun expression, thereby, activating EMT to promote tumor invasion in CRC.

## Discussion

Tumor metastasis is the main cause of poor prognosis of CRC, thus, early predictive diagnosis of highly metastatic CRC will be helpful for the clinical treatment of patients [30, 31]. In our study, we have demonstrated that *GFRA1* hypomethylation accompanied with upregulation of GFRA1 in invasive CRC, and correlated with poor prognosis of patients. The targeted demethylation of *GFRA1* by dCas9-TET1CD and specific gRNA promoted proliferation, migration, and metastasis of CRC cells *in vitro* and *in vivo*. Demethylation of *GFRA1* induced EMT in CRC by promoting AKT phosphorylation and increasing the AKT expression in CRC cells. Collectively, our findings indicate that *GFRA1* demethylation promotes CRC invasion *via* inducing EMT, and *GFRA1* methylation can be used a biomarker for early diagnosis of highly metastatic CRC.

DNA methylation, one of the most important factors driving tumorigenesis, are recognized as diagnosis biomarker of various cancers [6, 32, 33]. Previous studies have demonstrated the *GFRA1* methylation level was significantly decreased in gastric carcinoma with lymph/distant metastasis. Compared with patients with *GFRA1* high methylation, those with *GFRA1* low methylation had a poor relapse-free survival [5, 34]. These results indicated that GFRA1 promotes the metastasis in gastric carcinoma. In contrast, in hepatocellular carcinoma, the higher expression of GFRA1 was significantly correlated with good prognosis of patients [35]. In the present study, we revealed that *GFRA1* has lower methylation modification and higher expression in CRC invasive tumors as compared to non-invasive tumors. It also had been found that GFRA1 mRNA expression can be modulated by *GFRA1* methylation. Moreover, we noted CRC patients with GFRA1 high expression had poor overall survival and disease-free survival. Collectively, our results indicating that *GFRA1* methylation may have diagnostic potential for high metastatic CRC.

The accurate detection of DNA methylation location is essential for the understanding of regulating function of DNA methylation on the gene expression and cancer biological behaviors. [36–38]. The *GFRA1* methylation was significantly lower in CRC primary tumors with invasion, indicated that GFRA1 gene regions has the potential as a methylation biomarker for diagnosis and prediction of highly metastatic CRC. However, identification of a sequence that would be suitable as a methylation biomarker in GFRA1 gene remains unclear. For this reason, we further perform a functional analysis of the GFRA1 gene all-long using 450k methylation array data from TCGA. Results show that ten sequences in GFRA1 gene TSS region has lower methylation modifications in tumors with invasion compared to tumors without invasion. Among them, compared to tumors without invasion, the methylation level of the sequences downstream TSS is more obvious than of the sequences upstream of TSS in tumors with invasion. This phenomenon may be caused by different function of methylation modification [39]. Besides, ROC curve analysis suggests that these sequences have diagnostic value for CRC metastasis risk assessment and prediction; it is pretty helpful for metastatic CRC risk-tailored early diagnostic and primary treatment strategies. These sequences can also be combined with multiple indicators to improve the accuracy of diagnosis in clinical applications.

In terms of functionality, GFRA1 gene is involved in neurodevelopment [20, 21, 40] and spermatogenic stem cells differentiation [23, 41]. Meanwhile, ablation of GFRA1 may cause a Hirschsprung’s disease phenotype [42, 43]. In cancer promotion function, GFRA1 gene can encode GFRA1 protein to promote tumor progression by activating RET and downstream pathways in breast cancer and pancreatic cancer [26, 27, 44]. Furthermore, GFRA1 gene can also guide the generation of circRNA to regulate microRNA as an oncogene in breast cancer and ovarian cancer [45, 46]. These results explain that GFRA1 can perform multiple functions in a tumor. According to the hypermethylation of *GFRA1* in CRC, we chose to endogenously upregulate gene expression by targeted demethylation of *GFRA1*, instead of introducing an overexpression plasmid, which would better represent the function of targeted genes in cells [29, 47, 48]. Owing to the undesirable effects caused by the global demethylation process, DAC and AZA faced the serious challenges in experimental applications [49, 50]. In our study, *GFRA1* methylation editing capability to efficiently address the causal-effect relationships of *GFRA1* methylcytosine epigenetic in CRC invasion. Moreover, these results for off-target predictions also confirmed the specificity of dcas9-TET1CD-gGFRA1-5 and dcas9-TET1CD-gGFRA1-6 on demethylation of the GFRA1 promoter. *In vitro* experiments indicated that the migration and invasion of CRC cells were promoted by targeted demethylation of *GFRA1*. Meanwhile, more lung metastasis nodes were observed in the groups with *GFRA1* hypomethylation. However, we failed performing GFRA1 knockdown experiments due to no CRC cell line was identified with high endogenous GFRA1 expression. Importantly, our results indicated that *GFRA1* demethylation induced EMT in CRC cells and GFRA1 mRNA expression are highly correlated with expression of EMT-related factors in CRC tissues. These results suggested that GFRA1 promote invasion and metastasis through inducing EMT in CRC cells, which would expand the understanding of GFRA1 function.

Based on the inducing role of GFRA1 on EMT, Pathway Commons analysis was employed to determine the potential downstream targets of GFRA1. We found that GFRA1 is associated with AKT phosphorylation and c-Jun expression, which induce EMT in several cancers [51, 52]. Further results suggested that *GFRA1* demethylation promotes AKT phosphorylation and upregulates p-c-Jun and c-Jun expression. Furthermore, treatment of CRC cells with specific inhibitors targeting AKT or JNK could efficiently attenuate EMT mediated by demethylation of *GFRA1* in CRC cells. These findings suggested that GFRA1 induces EMT via AKT and c-Jun pathway. Meanwhile, AKT and JNK inhibitors would be an efficient therapy strategy for the CRC patients with *GFRA1* hypomethylation.

In summary, our findings indicate that GFRA1 functions as a tumor promoting factor to promote CRC invasion through inducing EMT. Methylation of *GFRA1* in CRC tissues can be a biomarker for diagnosis of highly metastatic CRC.

## Materials and methods

### Ethics statement

The CRC patient samples were obtained from the West China Hospital (Chengdu, P.R. China) with written informed consent, and the study was approved by West China Hospital of Sichuan University Biomedical Research Ethics Committee (S1 Text, Number: 2018(280)). Data including clinical information was acquired from the medical records. All mouse experimental procedures were approved by the Institutional Animal Care and Use Committees of Stake Key Laboratory of Biotherapy, Sichuan University (S2 Text, Number: 20181107004).

### Bioinformatic analysis

Expression profiles of mRNA, Illumina Human Methylation 450 K array data (β-value), and clinical information of CRC patients were downloaded from The Cancer Genome Atlas (TCGA v19.0; http://cancergenome.nih.gov/) database. β-value was used to measure the percentage of methylation [53–55]. β-value is defined as:

$$\beta =\frac{\text{max}(\text{methylated},0)}{\text{max}(\text{methylated},0)+\text{max}(\text{unmethylated},0)+\alpha }$$

Methylated and unmethylated are the intensity value from methylated and unmethylated probes and α is a constant offset (by default, α = 100). β-values range from 0 (completely unmethylated) to 1 (completely methylated).

The TCGA-CRC dataset contained a total of 521 CRC samples included 41 cases of paracancerous tissues and 480 cases of tumor tissues. The age of the samples is from 31 to 90 with the median age of 68. Numbers of female and male patients is 225 and 255, respectively. Differential expression and methylation level analysis was performed on the TCGA-CRC dataset using Limma package in R (version 3.5.1) software (https://www.r-project.org/). Wilcoxon rank-sum test was used to compare the difference of the expression of RNA-seq and methylation data between tumor tissues and adjacent tissue. A false discovery rate (FDR) <0.05 and |log_2_ fold change (FC)| >1 was set as the criteria for screening differentially expressed and methylation genes. Comparisons between tumor invasion (lymphatic and venous invasion) and no invasion were tested by two-tailed Student’s t-test, p < 0.05 was considered statistically significant.

GFRA1 gene CpG sites methylation level in CRC cell lines were downloaded from Cancer Cell Line Encyclopedia (CCLE 2019; https://portals.broadinstitute.org/ccle). GFRA1 related pathway commons network data were downloaded from the Public Pathway and Interactions Database [56] (2019 update; http://www.pathwaycommons.org/). Cytoscape software (version 3.7.0) was used to analyze functional pathways.

### DNA methylation analyses

The DNA extraction was performed using Tissue & Cell Genomic DNA Purification Kit (GeneMark #DP201). DNA and methylation quality analyses identified 75 CRC samples and 17 paracancerous samples suitable for the methylation study. To verify the results of methylation analysis of GFRA1 gene based on TCGA data, sequence (including parts of cg25617725 and cg12087643 sequence) upstream of the GFRA1 gene TSS was selected for time-of-flight mass spectrometry to determine methylation modification levels.

### Cell culture and plasmid transfection

Human colorectal cancer cells, HCT116, SW480, HT29, and RKO were maintained in Dulbecco’s modified Eagle’s medium (Gibco) supplemented with 10% fetal bovine serum (Gibco, MA, USA). All cell lines were maintained at 37°C with 5% CO_2_. The empty plasmid pPlatTET1-gRNA (Addgene plasmid: 82559) and GFRA1-targeted demethylation plasmid from pPlatTET1-gRNA1 to pPlatTET1-gRNA8 (the sequences of gRNA are displayed in Table S3) were transfected into HCT116 and SW480 cells using Lipofectamine 3000 reagent (Invitrogen #2024201, MA, USA), according to the manufacturer’s instructions. Successful transfection was verified as green fluorescence under fluorescence microscope. G418 (Sigma-aldrich #A1720, MA, USA) with a concentration of 2 g/L for 48 h was added for screening. After that, the HCT116 and SW480 cells transfected with the plasmid were used in subsequent experiments.

### Murine experimental lung metastasis experiments

Female BALB/c nude mice at 6 weeks of age were purchased from HFK Bioscience (Beijing, P.R. China). All mice were bred in the Animal Experimentation Unit (Sichuan University, Chengdu, China). Twenty one mice were randomly divided into three groups. Then, HCT116-pPlatTET-gRNA cells (control cells), HCT116-pPlatTET-gGFRA1-5 cells, and HCT116-pPlatTET-gGFRA1-6 cells (5×10^5^ cells/ mouse) were injected into the tail vein of each mouse. Mice were anaesthetised by intraperitoneal injection of 1% sodium pentobarbital(100μl/ mouse). All mice were sacrificed after one month and the lung tissues were removed for histological examination. After that, the mice lungs were fixed in 4% PFA for 48 h and embedded in paraffin, 5 μm sections were cut, and stained with hematoxylin and eosin (H&E).

### Reverse transcription and quantitative PCR (qRT-PCR)

CRC cells and CRC patient samples were harvested using Trizol (Ambin #15596026, CA, USA), according to manufacturer’s instructions. RNA was converted to cDNA using PrimeScript RT reagent Kit with gDNA Eraser (Perfect Real Time) (Takara #RR047B, Tokoyo, Japan). Quantitative PCR reactions were prepared with TB green Premix EX TaqII (Takara #RR820A, Tokoyo, Japan), and performed in Light Cycler 96 System (Roche, Switzerland). Primer sequence information for RT-qPCR is listed in S5 Table.

### Western blotting

CRC cells and patient samples were lysed using RIPA lysis buffer containing proteinase inhibitor (1:100, Invitrogen #87785), and was subjected to immunoblot analysis. Mouse anti-GFRA1 (1:1000, Santacruz #sc-271546, MA, USA), rabbit anti-VIM (1:1000, Cell Signaling #5741, CA, USA), rabbit anti-N-cad (CDH2) (1:1000, ServiceBio #GB11135, Wuhan, China), rabbit anti-AKT (1:1000, Cell Signaling #4691, CA, USA), rabbit anti-p-AKT (1:1000, Cell Signaling #4060, CA, USA), rabbit anti C-Jun (1:1000, Cell Signaling #6195, CA, USA), and rabbit anti p-C-Jun (1:1000, Cell Signaling #3270, CA, USA) antibodies were used. Decitabine (DAC), MK-2206 2HCl and SP600125 was purchased from Selleck.

### Immunohistochemistry and immunofluorescence

For immunohistochemistry (IHC), patient tissues were fixed in 4% Paraformaldehyde, embedded in paraffin, and then cut into 5 μm thick sections for IHC staining. The slides were blocked with non-immune goat serum and incubated with anti-mouse GFRA1 antibody (1:200 Santacruz #sc-271546) for 24 h at 4°C. Then follow the instructions (ZSGB-BIO #SP-9002, Beijing, China).

For immunofluorescence, the transfected HCT116 cells and SW480 cells grown on the cover glass in 24 well plates were fixed with 4% paraformaldehyde (PFA) for 20 min and permeabilized with 0.2% Triton X-100 for 15 min at room temperature (22–25°C). Then, the cells were incubated with the desired primary antibodies in PBS for 24 h at 4°C, washed with PBS 3 times, and then incubated with fluorescent secondary antibodies away from light at room temperature (22–25°C). After that, the nuclei were stained with DAPI. The fluorescence intensity and absorbance were measured at 555 nm (Invitrogen #A21428, MA, USA) and 488 nm (Invitrogen #A11001, MA, USA), respectively.

### Bisulfite sequencing PCR

Bisulfite Sequencing (BSP-seq) of all bisulfite converted genomic DNA samples was performed with DNA Bisulfite Conversion Kit (TIANGEN # DP215-02), according to the manufacturer’s instructions. The primer sequences that were used are as follows:

GFRA1-F1 5´-GTTTTAGGAGAGAGGTAGAGATTG-3´

GFRA1-R1 5´-AATACTACCAAACACACACACTCT-3´

GFRA1-F2 5´-GCGTATTTTAGGATCGTCG-3´

GFRA1-R2 5´-CAAAACACGCAATATTCTACA-3´

PCR reactions were done using EpiTaq HS (TaKaRa #R110Q, Tokoyo, Japan). After purifying the PCR product by GEL Extraction Kit (OMEGA # D2500-01), this DNA fragment was subsequently cloned into pClone007 Simple Vector (TSINGKE #TSV-007S, Beijing, China) for sequencing. Methylation levels of CpG site in GFRA1 gene fragments were analyzed by QUMA (http://quma.cdb.riken.jp/).

### Cell proliferation and clonogenic assay

For CCK8 cell proliferation assay, HCT116 (5000 cells/ per well) and SW480 (10000 cells/ per well) cells were plated in four 96 well plates with three repeated wells assigned for each group. Absorption values were measured 2 h after adding CCK8 reagent (DOJINDO #CK04), every 24 h. The optical density (OD) was measured at 450 nm.

For cell clonogenic assay, the transfected HCT116 cells (1000 cells/ well) and SW480 cells (5000 cells/ well) were replated in the 6 well plates, and cultured in complete medium for 2 weeks. Then, the cells were stained with crystal violet and the individual colonies were counted.

### Migration and invasion assays

Cell culture insert transparent PET Membrane, 24 Well, 8.0 um pore size (Falcon #353097) were used for the migration and invasion assays. A total of 600 μl of culture medium containing 20% FBS (Gibco, MA, USA) was added to the lower chamber. In the migration assay, 1×10^5^ GFRA1-hypomethylation HCT116 or SW480 cells and their respective control cells in 100 μl serum-free medium were plated on the uncoated insets, and were incubated for 48 h. In the invasion assay, the insets were coated with 60 μl of 1:10-diluted matrigel (BD Biosciences, MA, USA), and 1.5 × 10^5^ cells in 100 μl serum-free medium were plated on the insets for an incubation period of 48 h. After that, the cells attached to the membrane were fixed with 4% PFA for 20 min, stained with 5% crystal violet (Beyotime #C0121, Beijing, China) 10 min, and counted at 100x magnification.

### Statistical analysis

Statistical analysis was performed using GraphPad software (version 8.00) and p < 0.05 was considered statistically significant. Comparisons between the two groups were analyzed by two-tailed Student’s t-test. Receiver operating characteristic (ROC) curve analysis was used to test the effectiveness of the methylation level of the GFRA1 DNA probe for the diagnosis of invasive CRC tumors. Correlation analysis was evaluated by Pearson’s correlation test. Survival was determined by log-rank (Mantel-Cox) test. Measurement data are mean ± SD from at least three independent experiments.

## Supporting information

S1 Fig Methylation of *GFRA1* can regulate gene expression. **A** Venn diagram showing the number of genes with methylation modification and significantly reduced expression levels from TCGA CRC RNA-seq data and methylation data (FDR < 0.05, Wilcoxon rank-sum test). **B** Heat map showing associated with the expression profile of 65 genes in normal and tumor tissues from S1A Fig, gene expression levels are represented by the color of the heat map (red means high expression, green means low expression). **C** Q-PCR analysis showing the expression level of GFRA1 in HCT116 and SW480 cells after treatment gradient concentration DAC for 48h. (**p < 0.01, ***p < 0.01, ****p < 0.0001 student t-test). **D** Western blot analyses display the effects of DAC on the protein expression of GFRA1 in HCT116, SW480, HT29 and RKO. **E** Immunofluorescence assays display localization and expression of GFRA1 in HCT116 and SW480 cells treated with DAC. Scale bars, 100 μm. **F** Immunohistochemical analysis of GFRA1 expression in primary tumor tissue from HXCRC cohort TMA. Scale bars, 100 μm.
(TIF)

S2 Fig Methylation levels are significantly associated with gene expression and tumor invasion in the downstream sequences of *GFRA1* TSS. **A-G** Detailed description of seven methylation sequences upstream of GFRA1 gene TSS from (Fig 2C), including correlation between gene expression and DNA methylation, Variety in DNA methylation levels at different tumor stages and Differences in methylation levels between invasive and non-invasive tumors (*p < 0.05,**p < 0.01, ***p < 0.01, ****p < 0.0001 student t-test).
(TIF)

S3 Fig ROC curves for the diagnosis of CRC Lymphatic invasion by *GFRA1* TSS sequences. **A** ROC curves for cg13320291. **B** ROC curves for cg24792682. **C** ROC curves for cg06298519. **D** ROC curves for cg19485539. **E** ROC curves for cg17256532. **F** ROC curves for cg19236679. **G** ROC curves for cg03503087. **H** ROC curves for cg25617725.
(TIF)

S4 Fig *GFRA1* TSS CpG methylation level in CRC cell lines. **A** Heatmap visualization of GFRA1 gene CpG sites methylation level in CRC cells form CCLE database. (Those sites with no data in CRC cell lines were colored in grey). **B** BSP-seq showing the methylation level in HCT116 and SW480 cells (methylation CpG sites are shown as black dots, unmethylation CpG sites are shown as white dots). **C-D** The relative expression of genes at gGFRA1-5 and gGFRA1-6 off-target sites in SW480 cells (Student’s t-test). Data are mean ± SD.
(TIF)

S5 Fig Genes that do not show a correlation at the expression level with targeted removal of *GFRA1* methylation in CRC cells. **A** Gene co-expression analysis display the correlation between of GFRA1 and IGF1, FGF2, VEGFC, VEGFA, MMP7, CDH1, TWIST1, SNAI1, SNAI2 (Pearson correlation test). **B-C** Q-PCR showing the effects of IGF1, FGF2, VEGFC, SNAI2 expression in GFRA1 demethylation HCT116 and SW480 cells (student t-test).
(TIF)

S1 Table 450k probe sequences related to CRC invasion. (DOCX)

S2 Table gRNA sequences about GFRA1 gene targeted demethylation. (DOCX)

S3 Table gRNA5 sequence predicted off-target sites. (DOCX)

S4 Table gRNA6 sequence predicted off-target sites. (DOCX)

S5 Table q-PCR primer sequences. (DOCX)

S1 Text Human ethics certification. (PDF)

S2 Text Animal experiment ethics certification. (PDF)
