# Supplementary figures and images for: Hypomethylation of GDNF family receptor alpha 1 promotes epithelial-mesenchymal transition and predicts metastasis of colorectal cancer
Source: PLoS Genet. 2020 Nov 11;16(11):e1009159. doi: 10.1371/journal.pgen.1009159 (PMC7682896; doi:10.1371/journal.pgen.1009159)

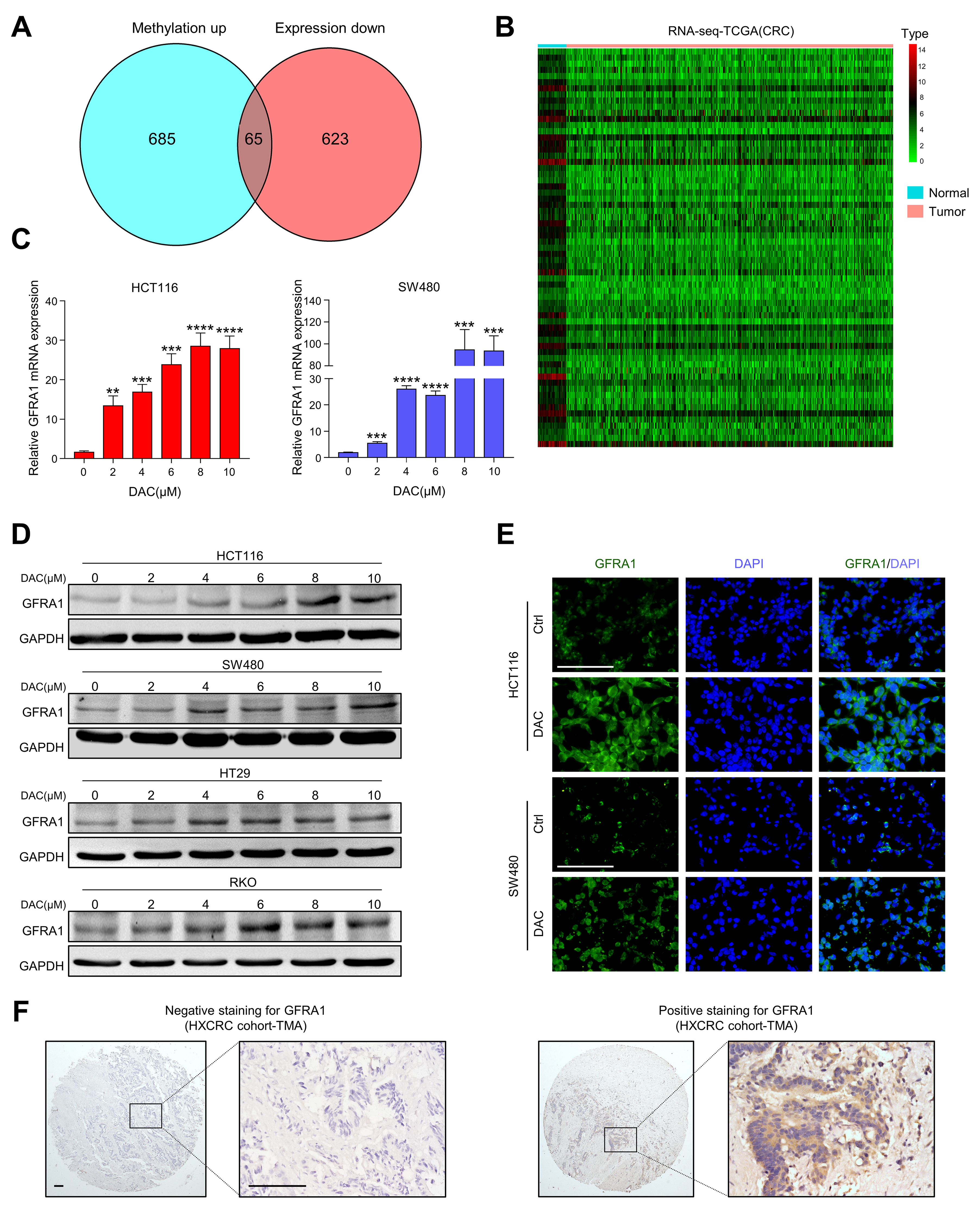

Supplement: S1 Fig — A Venn diagram showing the number of genes with methylation modification and significantly reduced expression levels from TCGA CRC RNA-seq data and methylation data (FDR < 0.05, Wilcoxon rank-sum test). B Heat map showing associated with the expression profile of 65 genes in normal and tumor tissues from S1A Fig, gene expression levels are represented by the color of the heat map (red means high expression, green means low expression). C Q-PCR analysis showing the expression level of GFRA1 in HCT116 and SW480 cells after treatment gradient concentration DAC for 48h. (**p < 0.01, ***p < 0.01, ****p < 0.0001 student t-test). D Western blot analyses display the effects of DAC on the protein expression of GFRA1 in HCT116, SW480, HT29 and RKO. E Immunofluorescence assays display localization and expression of GFRA1 in HCT116 and SW480 cells treated with DAC. Scale bars, 100 μm. F Immunohistochemical analysis of GFRA1 expression in primary tumor tissue from HXCRC cohort TMA. Scale bars, 100 μm. (TIF) [file pgen.1009159.s001.tif]

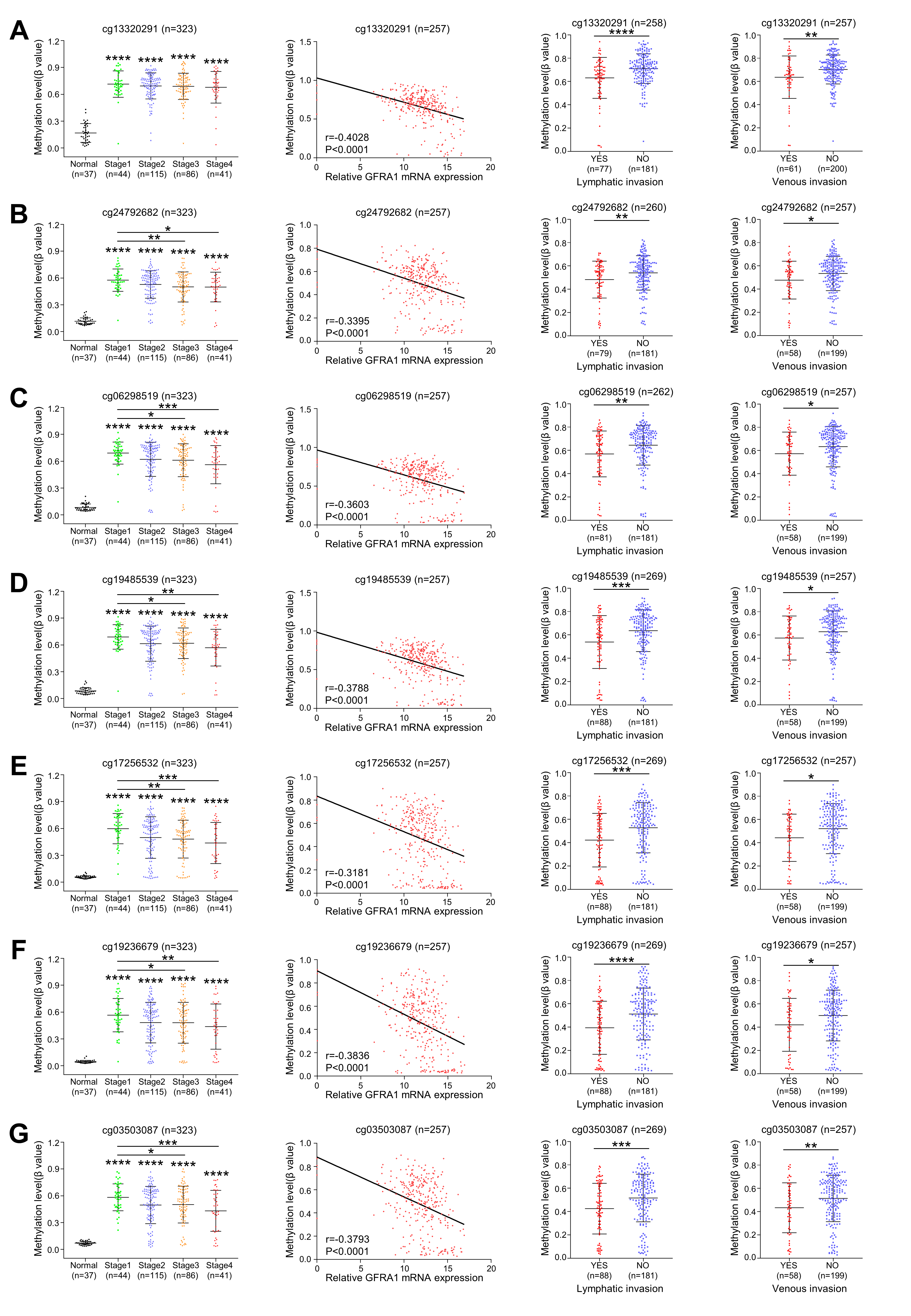

Supplement: S2 Fig — A-G Detailed description of seven methylation sequences upstream of GFRA1 gene TSS from (Fig 2C), including correlation between gene expression and DNA methylation, Variety in DNA methylation levels at different tumor stages and Differences in methylation levels between invasive and non-invasive tumors (*p < 0.05,**p < 0.01, ***p < 0.01, ****p < 0.0001 student t-test). (TIF) [file pgen.1009159.s002.tif]

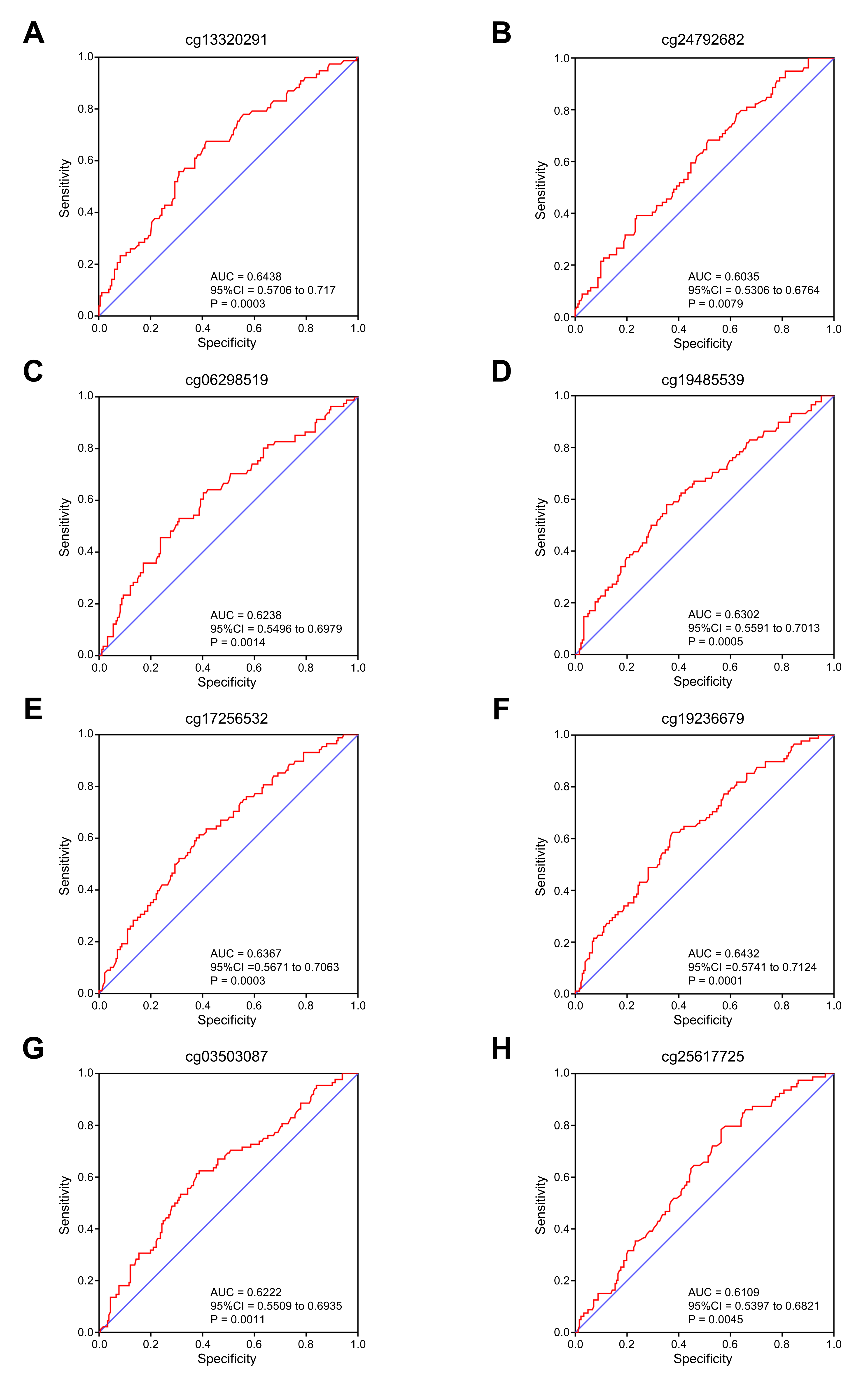

Supplement: S3 Fig — A ROC curves for cg13320291. B ROC curves for cg24792682. C ROC curves for cg06298519. D ROC curves for cg19485539. E ROC curves for cg17256532. F ROC curves for cg19236679. G ROC curves for cg03503087. H ROC curves for cg25617725. (TIF) [file pgen.1009159.s003.tif]

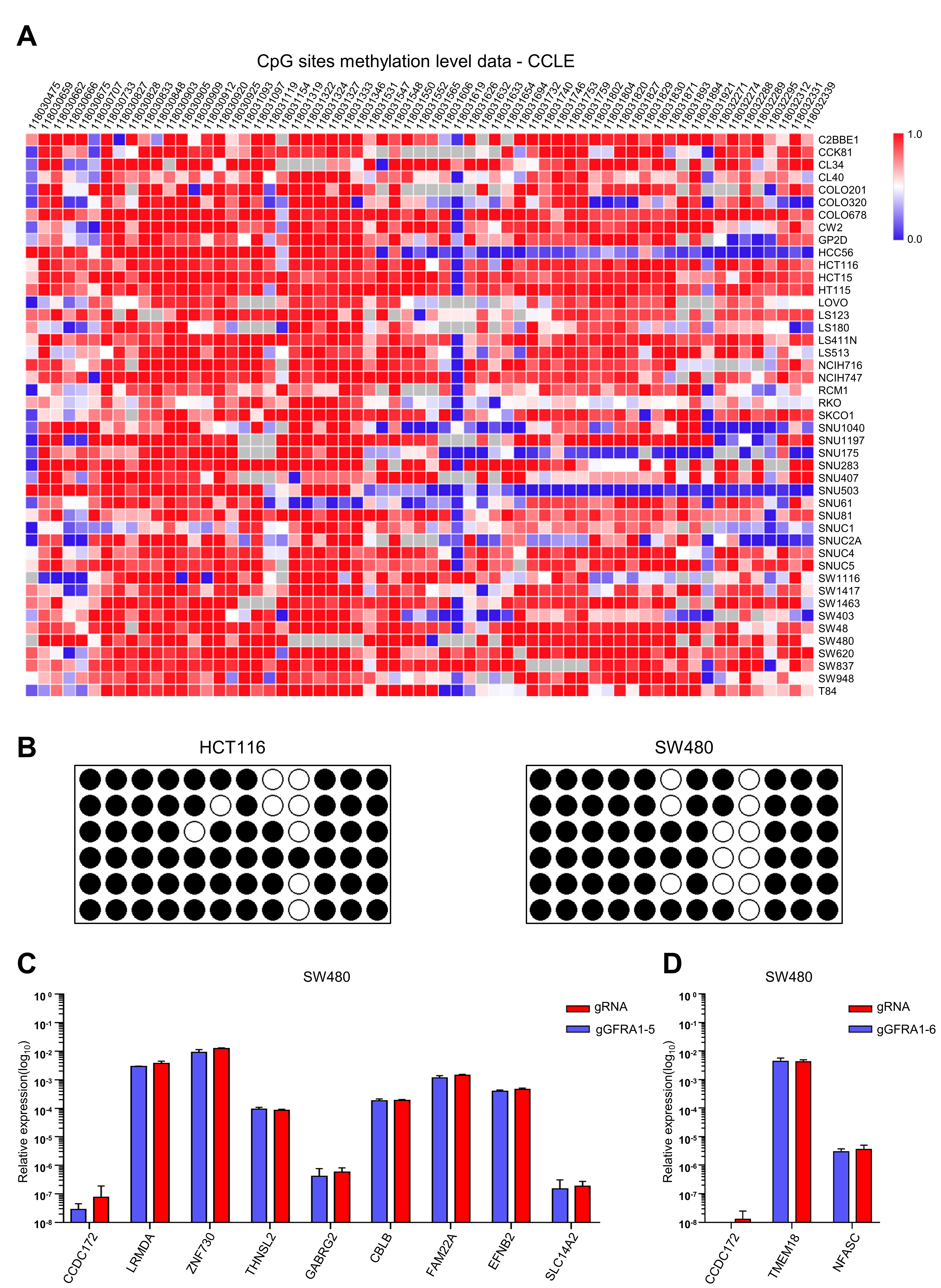

Supplement: S4 Fig — A Heatmap visualization of GFRA1 gene CpG sites methylation level in CRC cells form CCLE database. (Those sites with no data in CRC cell lines were colored in grey). B BSP-seq showing the methylation level in HCT116 and SW480 cells (methylation CpG sites are shown as black dots, unmethylation CpG sites are shown as white dots). C-D The relative expression of genes at gGFRA1-5 and gGFRA1-6 off-target sites in SW480 cells (Student’s t-test). Data are mean ± SD. (TIF) [file pgen.1009159.s004.tif]

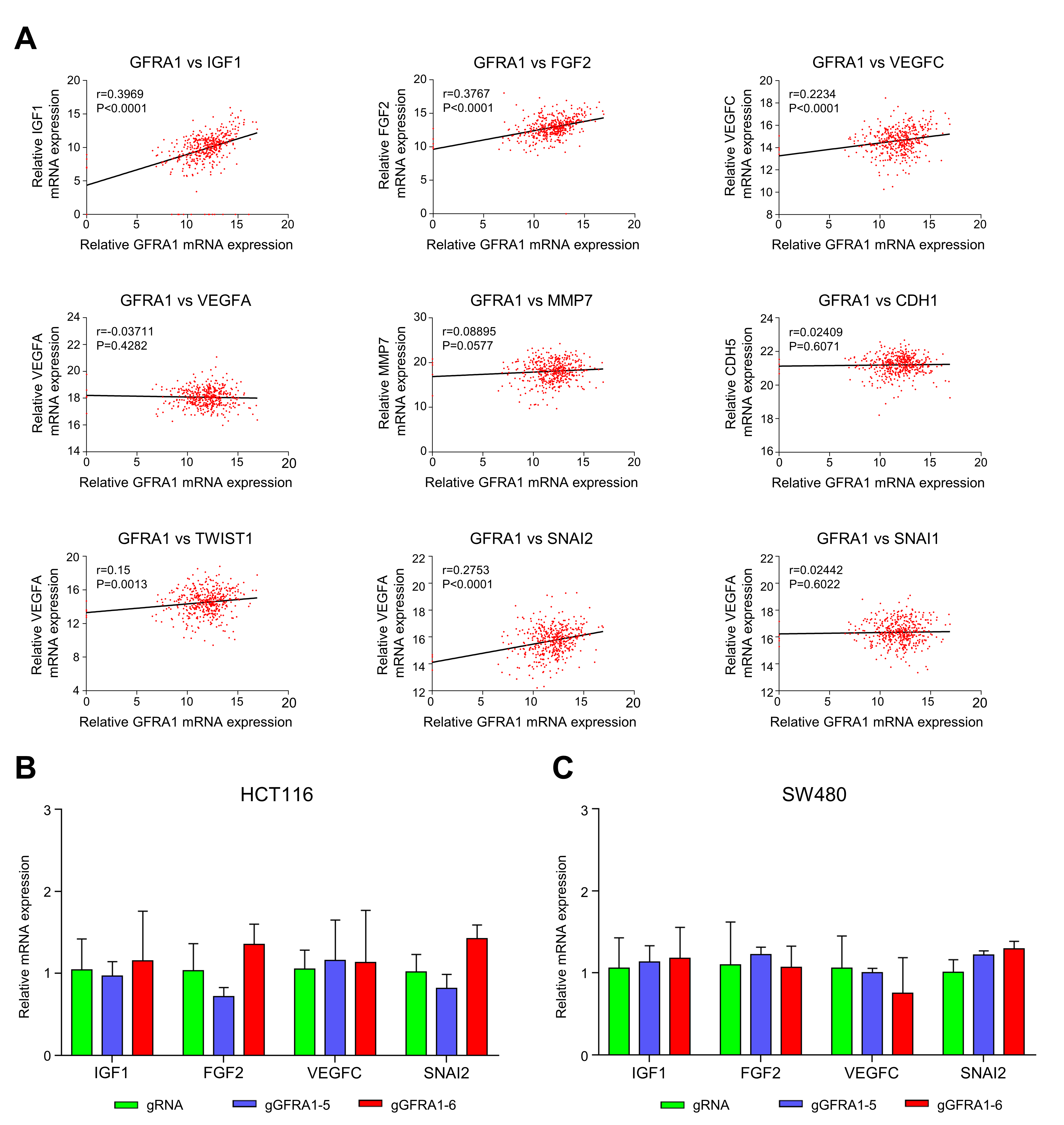

Supplement: S5 Fig — A Gene co-expression analysis display the correlation between of GFRA1 and IGF1, FGF2, VEGFC, VEGFA, MMP7, CDH1, TWIST1, SNAI1, SNAI2 (Pearson correlation test). B-C Q-PCR showing the effects of IGF1, FGF2, VEGFC, SNAI2 expression in GFRA1 demethylation HCT116 and SW480 cells (student t-test). (TIF) [file pgen.1009159.s005.tif]
