# Supplementary material for: Hypomethylation of GDNF family receptor alpha 1 promotes epithelial-mesenchymal transition and predicts metastasis of colorectal cancer
Source: PLoS Genet. 2020 Nov 11;16(11):e1009159. doi: 10.1371/journal.pgen.1009159 (PMC7682896; doi:10.1371/journal.pgen.1009159)
Supplement: S1 Table — (DOCX) [file pgen.1009159.s006.docx]

**S1 Table. 450k probe sequences related to CRC invasion**

| **Target ID** | **Sequence** |
| --- | --- |
| cg13320291 | CTCCTCGGAAAGCGCCTGAGTTTCAGCCGGGCGGAGCGGGTGGCTGGAGCCGGGGGTGGCCCGGGAGCCCGGGTCGTTGCTCAGGCCAGGATGCCCCGGGG |
| cg24792682 | CTTTCACTGGATGGAGCTGAACTTTGGGCGGCCAGAGCAGCACAGCTGTCCGGGGATCGCTGCACGCTGAGCTCCCTCGGCAAGACCCAGCGGCGGCTCGG |
| cg06298519 | GGTGGGGGTGGGTCCTCACCCCGGTGTTGGAAATTCCCCAAAGGCGGGAACGGGGGAGGGGAGAGGGTTCTGTGGGGGGAGTCTCCGGCGCTCTCCGCTCT |
| cg19485539 | GACCTGGGTGGGGGTGGGTCCTCACCCCGGTGTTGGAAATTCCCCAAAGGCGGGAACGGGGGAGGGGAGAGGGTTCTGTGGGGGGAGTCTCCGGCGCTCTC |
| cg17256532 | AGGACCCACCCCCACCCAGGTCGGGGTGCATGTGCGTGTTTTCCAGGGGCCGCTGACACGGGGATGGAGGTGAGGGCTGGAGAGGTCTGAAGAGGGTTCCT |
| cg19236679 | GGGTAGGGGGTAGGGGCGCAGGAACCGGGTTTCAGCCCCAGTCGGGACATCGGTTCCCCCTGGCTTGGCGGCCTCTGGAGGGAGACGAGTCCCGGGGAGAA |
| cg03503087 | CAGGTTGGTGGCATTTGGAGTTTTTTTCGTTCTGTTTGGGAACCCATTGCCGCTTCCCGTCCCTCTTCCCCACTCCTTCTTCCCCCCTCCTCCGGCCACCC |
| cg23898073 | CAGGAGCAGGCCGAGGGTCCTCTGGCCAGAAGAAATCTGGCCTCGGAACACGCCATTCTCCGCGCCGCTTCCAATAACCACTAACATCCCTAACGAGCATC |
| cg25617725 | ACGGGCACAAGGGCTCTTTCTTCTTAGGGCAATCGAGCCAGACCCTTCTTCGGAACACAAAGTTTTCCCCCACGTCACTAGTGGCTGTGATTCAAGACTTG |
| cg12087643 | GTGGCTGTGATTCAAGACTTGGTGCTTTCAAGACACACTCAGACCCAGGACGCTGGAGCGGCGATCCTGGGATGCGCCCATCCGTTCGTCCACGCCAGCGC |
