# Supplementary material for: Hypomethylation of GDNF family receptor alpha 1 promotes epithelial-mesenchymal transition and predicts metastasis of colorectal cancer
Source: PLoS Genet. 2020 Nov 11;16(11):e1009159. doi: 10.1371/journal.pgen.1009159 (PMC7682896; doi:10.1371/journal.pgen.1009159)
Supplement: S2 Table — (DOCX) [file pgen.1009159.s007.docx]

**S2 Table. gRNA sequences about GFRA1 gene targeted demethylation**

| **gRNA ID** | **Oligonucleotides** |
| --- | --- |
| gGFRA1-1 | GTGTCACATTTTCGCGACAGA |
| gGFRA1-2 | GCGTAGGATGTCCCCCGGGTT |
| gGFRA1-3 | GTAGGATGTCCCCCGGGTTT |
| gGFRA1-4 | GCGCCAGACAATGGGCCGCCG |
| gGFRA1-5 | GGCCCATTGTCTGGCGTGAT |
| gGFRA1-6 | GCGGGGCACCGAAGTCTACAC |
| gGFRA1-7 | GCTGGCGTGGACGAACGGAT |
| gGFRA1-8 | GCGTGTCCTGTGCGCGAGTAC |
