# Supplementary material for: Hypomethylation of GDNF family receptor alpha 1 promotes epithelial-mesenchymal transition and predicts metastasis of colorectal cancer
Source: PLoS Genet. 2020 Nov 11;16(11):e1009159. doi: 10.1371/journal.pgen.1009159 (PMC7682896; doi:10.1371/journal.pgen.1009159)
Supplement: S3 Table — (DOCX) [file pgen.1009159.s008.docx]

**S3 Table. gRNA5 sequence predicted off-target sites**

| **Search result** | **Chr Position** | **Strand** | **Mismatch** | **Gene** | **Score** |
| --- | --- | --- | --- | --- | --- |
| GGCCCATTGTCTGGCGTGATAGGT | Chr10:116274362-116274385 | - | 1 | CCDC172 | 40.7 |
| GGCCCATTGTCTGGCGTGATAG | Chr10:116274364-116274385 | - | 1 | CCDC172 | 40.51 |
| GGCCCATTGTCTGGCATAGATGTG | Chr10:76455715-76455738 | + | 2 | LRMDA | 27 |
| GGCCCATTATCTGGGTGATGTG | Chr19:23018003-23018024 | - | 2 | ZNF730 | 22.76 |
| GGCCCATGGTCTGGGTGATGGC | Chr2:88185367-88185388 | - | 2 | THNSL2 | 22.68 |
| GGCCATTGTCTGACGTGATCTG | Chr5:162538240-162538261 | + | 2 | GABRG2 | 21.96 |
| GGCCCATTGTCAGGTGTGAAAGG | Chr3:106549146-106549168 | - | 3 | CBLB | 8.7 |
| AGCCCATTGTCTGGCATGTGGG | Chr12:109732469-109732490 | + | 2 | FAM22A | 7.93 |
| GGCCCATCTGTCTGGGGTGCTGGG | Chr13:106454551-106454574 | + | 2 | EFNB2 | 7.87 |
| GGCCCATTGCTGGCTTAATTGG | Chr18:45686631-45686652 | - | 2 | SLC14A2 | 7.31 |
