# Supplementary material for: Hypomethylation of GDNF family receptor alpha 1 promotes epithelial-mesenchymal transition and predicts metastasis of colorectal cancer
Source: PLoS Genet. 2020 Nov 11;16(11):e1009159. doi: 10.1371/journal.pgen.1009159 (PMC7682896; doi:10.1371/journal.pgen.1009159)
Supplement: S4 Table — (DOCX) [file pgen.1009159.s009.docx]

**S4 Table. gRNA6 sequence predicted off-target sites**

| **Search result** | **Chr Position** | **Strand** | **Mismatch** | **Gene** | **Score** |
| --- | --- | --- | --- | --- | --- |
| CCGGGGCACCGAAGTCTACACTG | Chr10: 116274216-116274238 | + | 2 | CCDC172 | 40.61 |
| CCGGGGCACCGAAGTCTACACTGGG | Chr10: 116274216-116274240 | + | 1 | CCDC172 | 20.8 |
| GCGGGGGCACCGAAGTTCACACGGG | Chr2: 629076-629100 | + | 2 | TMEM18 | 5.11 |
| GGGGGCACCGAAGTTCACACGGG | Chr2: 629078-629100 | + | 2 | TMEM18 | 4.83 |
| GCTGGCCACCGAAGTCACACTGG | Chr1: 204995611-204995633 | - | 2 | NFASC | 3.13 |
| CCCGGGGCACCGAAGTCTACACTGG | Chr10: 116274215-116274239 | + | 2 | CCDC172 | 1.14 |
| CCCGGGGCACCGAAGTCTACACTGG | Chr10: 116274215-116274239 | + | 2 | CCDC172 | 1.12 |
| CCCGGGGCACCGAAGTCTACACTGG | Chr10: 116274215-116274239 | + | 2 | CCDC172 | 1.1 |
| CCCGGGGCACCGAAGTCTACACTGG | Chr10:116274215-116274239 | + | 2 | CCDC172 | 1.08 |
| CCCGGGGCACCGAAGTCTACACTGG | Chr10:116274215-116274239 | + | 1 | CCDC172 | 0.93 |
