# Supplementary material for: Hypomethylation of GDNF family receptor alpha 1 promotes epithelial-mesenchymal transition and predicts metastasis of colorectal cancer
Source: PLoS Genet. 2020 Nov 11;16(11):e1009159. doi: 10.1371/journal.pgen.1009159 (PMC7682896; doi:10.1371/journal.pgen.1009159)
Supplement: S5 Table — (DOCX) [file pgen.1009159.s010.docx]

**S5 Table. q-PCR primer sequences**

| **Name** | **primer sequence** |
| --- | --- |
| H-TGFB2-F | GCGACGAAGAGTACTACGCC |
| H-TGFB2-R | GGCATCAAGGTACCCACAGA |
| H-GFRA1-F | TCCAATGTGTCGGGCAATAC |
| H-GFRA1-R | GGAGGAGCAGCCATTGATTT |
| H-MMP2-F | CAAGTTCCCCGGCGATGTC |
| H-MMP2-R | GCACAAACAGGTTGCAGCTC |
| H-MMP9-F | GTACTCGACCTGTACCAGCG |
| H-MMP9-R | AGAAGCCCCACTTCTTGTCG |
| H-VIM-F | TCACCTGTGAAGTGGATGCC |
| H-VIM-R | ACGAAGGTGACGAGCCATTT |
| H-CDH2-F | AGGCTTCTGGTGAAATCGCA |
| H-CDH2-R | AGAGGCTGTCCTTCATGCAC |
| H-TCF4-F | TGCTTGCATACATTGCCAGT |
| H-TCF4-R | CTCTGGTGGCAACCCTGTAA |
| H-ZEB2-F | AACAAGCCAATCCCAGGAG |
| H-ZEB2-R | ACCGTCATCCTCAGCAATATG |
| H-ZEB1-F | GATGACCTGCCAACAGACCA |
| H-ZEB1-R | CCCCAGGATTTCTTGCCCTT |
| H-GAPDH-F | GGGAGCCAAAAGGGTCATCA |
| H-GAPDH-R | TGATGGCATGGACTGTGGTC |
| H-IGF1-F | GCTCTTCAGTTCGTGTGTGGA |
| H-IGF1-R | GCCTCCTTAGATCACAGCTCC |
| H-FGF2-F | TGCTCAGCAGTCACCATAGC |
| H-FGF2-R | CTTGAGGTGGAAGGGTCTCC |
| H-VEGFC-F | GCTTCTTCTCTGTGGCGTGT |
| H-VEGFC-R | TTTGCTTGCATAAGCCGTGG |
| H-SLUG-F | CCAAGCTTTCAGACCCCCAT |
| H-SLUG-R | TGCAGCTGCTTATGTTTGGC |
| H-CCDC172-F | GAGGAGAGTCGCCGTTTGAT |
| H-CCDC172-R | GCTTTCAAAAGCTTAGATTCCAGC |
| H-LRMDA-F | GGTGCGTGGAACTCAAGTGT |
| H-LRMDA-R | GCGAAATGTCCACAGTCCCT |
| H-ZNF730-F | ACCCTGGAAGCTGTGAAGTG |
| H-ZNF730-R | AGGTCTGGCTTTGAGACAGC |
| H-FAM222A-F | TGTCTGCAGAGGACCCAGAA |
| H-FAM222A-R | ATAGGCGTCCAGTTCTGCTG |
| H-EFNB2-F | TGTGAGAAGGGACTCCGTGT |
| H-EFNB2-R | TTAGAGTCCACTTTGGGGCA |
| H-TMEM18-F | CTCTGTCAGCTCTTTCCCCG |
| H-TMEM18-R | TCTGTAGCTTCGGGAGGACA |
| H-NFASC-F | TATGGAACGTCACGGTGCTC |
| H-NFASC-R | CTGGGCCTTAACTGGGACAG |
| H-THNSL2-F | CAAGGAACTGCACAGCAAGTG |
| H-THNSL2-R | CAGCACCCATCACACTCCATT |
| H-SLC14A2-F | TGCTGTTACAAGCCATCCCT |
| H-SLC14A2-R | AGAGGCCTGTGTAGATGGTCT |
| H-CBLB-F | AGATGGTTCACAGGCACCAG |
| H-CBLB-R | CCCATGGGGTTTTCTGTGGT |
| H-GABRG2-F | GACAATGACCACCCTCAGCA |
| H-GABRG2-R | CCTTGCTTGGTTTCCGGTTG |
