# Supplementary material for: Hypomethylation of GDNF family receptor alpha 1 promotes epithelial-mesenchymal transition and predicts metastasis of colorectal cancer
Source: PLoS Genet. 2020 Nov 11;16(11):e1009159. doi: 10.1371/journal.pgen.1009159 (PMC7682896; doi:10.1371/journal.pgen.1009159)
Supplement: S1 Text — (PDF) [file pgen.1009159.s011.pdf]

四川大学华西医院生物医学伦理委员会审查批件

2018年 审（280）号

科室（专业）： 胃肠外科	项目负责人姓名及职称: 周总光 教授	
项目名称	中国人群结直肠癌患者基因组变异研究	
研究方案	版本号:Version1.0	版本日期:2018.08.01
知情同意书	无	
招募广告:	无	

审查意见：

1. 研究者资质符合伦理要求。
2. 研究方案基本符合伦理要求。
3. 同意豁免知情同意书。

审查结果：■批准 □修改后批准 □修改后再审 □不批准 □暂停或者终止研究

持续审查频率： □3 个月/3months □6 个月/6months □1 年/1year ■不适用/NA

请遵循我国相关法律、法规和规章（《涉及人的生物医学研究伦理审查办法》等）以及WMA《赫尔辛基宣言》和CIOMS《人体生物医学研究国际道德指南》，遵循伦理委员会批准的方案和知情同意书开展临床试验（研究），保护受试者的健康与权利。

请遵循《人类遗传资源管理暂行办法》和《人类遗传资源采集、收集、买卖、出口、出境审批行政许可服务指南》，对我国人类遗传资源（例如生物样本等）及其相关数据信息采集和使用等仅限在我国境内，未经我国相关部门审批，人类遗传资源及其相关数据信息不能出境；若储存的生物样本作其他研究之用，必经华西医院生物医学伦理委员会审查同意。

在试验（研究）过程中，若变更主要研究者，对临床研究方案、知情同意书等的任何修改，请申请人提交修正案审查申请。

发生严重不良事件，请申请人及时提交严重不良事件报告；紧急报告之后，尽快提交详细的严重不良事件随访报告。

请递交年度和定期跟踪审查报告；当出现任何可能显著影响试验（研究）进行或增加受试者危险的情况时，请申请人及时向伦理专委会提交书面报告。

试验（研究）纳入了不符合纳入标准或符合排除标准的受试者，符合中止试验（研究）规定而未让受试者退出试验（研究），给予错误治疗或剂量，给予方案禁止的合并用药等没有遵从方案开展研究的情况；或可能对受试者的权益/健康、以及研究的科学性造成不良影响等违背伦理原则与规范的情况，请申办者/监查员/研究者提交违背方案报告。

申请人暂停或提前终止临床试验（研究），请及时提交暂停/终止试验（研究）报告。完成临床试验（研究），请申请人提交结题报告。

未经伦理审查批准,不能开展临床研究。

根据国际医学期刊编辑委员会（ICMJE）要求，所有在人体中和采用取自人体的标本进行的临床研究均应注册。研究者务必在临床研究开始前到中国临床研究注册中心注册，请使用我院公共帐号(请发邮件到临床研究管理部邮箱 hxlcyjglb@163.com 申请)登录以下网址进行临床研究注册：http://www.chictr.org.cn，临床研究项目注册成功后产生的唯一注册号请及时发送短信到伦理办公室，是伦理跟踪审查的必查项目。

单 位（章）： 四川大学华西医院 生物医学伦理委员会

主任委员（签名）：

2018年 8 月 21 日
