# Supplementary material for: Hypomethylation of GDNF family receptor alpha 1 promotes epithelial-mesenchymal transition and predicts metastasis of colorectal cancer
Source: PLoS Genet. 2020 Nov 11;16(11):e1009159. doi: 10.1371/journal.pgen.1009159 (PMC7682896; doi:10.1371/journal.pgen.1009159)
Supplement: S2 Text — (PDF) [file pgen.1009159.s012.pdf]

Sichuan University
17#, 3rd Section, Renmin South Road, Chengdu, Sichuan 610065 P.R.China

Certification

This is to certify that the animals use protocol listed below has been reviewed and approved by the Animal experimental ethics committee of State Key Laboratory of Biotherapy, Sichuan University. The Approval No. is 20181107004.

Protocol title: The study of the effects of GFRA1 demethylation on colorectal cancer metastasis *in vivo*

Applicant: Zhexu Dong

Principle Investigate (PI): Hongxin Deng

Department: Department of tumor biology

Species or Strains: BALB/c-nu

Quantity: BALB/c-nu (120)

Period of Protocol: 2018.12.2~2020.6.30

Animal experimental ethics committee

State Key Laboratory of Biotherapy

Sichuan University

2018.11.10
